# Use of Hospital Appointment Registration Systems in China: A Survey Study

**DOI:** 10.5539/gjhs.v5n5p193

**Published:** 2013-07-22

**Authors:** Weiping Yu, Xiaowen Yu, Hao Hu, Guimin Duan, Zi Liu, Yitao Wang

**Affiliations:** 1 Business School, Sichuan University, Chengdu, China; 2 State Key Laboratory of Quality Research in Chinese Medicine, Institute of Chinese Medical Sciences, University of Macau, Macao; 3 Chengdu University of Traditional Chinese Medicine, Chengdu, China; 4 West China Hospital, Sichuan University, Chengdu, China

**Keywords:** appointment registration system, hospital, patient, China, OAS: the on-site appointment system, 114: 114 telephone appointment system, 95533: telephone appointment system based on China Construction Bank's customer call center, CAS: clinical appointment system, BST: bank self-service terminal appointment system, WAS: web-based appointment system

## Abstract

**Background:**

Hospitals have expressed no knowledge of patients’ opinions regarding diversified appointment registration systems, despite efforts to develop novel appointment registration systems that assist patients and increase hospital efficiency. Therefore, the aim of this study was to investigate the use of diversified appointment registration systems and explore the factors influencing patients’ registration system choices.

**Methods:**

A survey study using a questionnaire was conducted in West China Hospital in February 2012. Outpatients were randomly selected from different hospital departments and the questionnaire was distributed and collected on-site.

**Results:**

Data from 1,009 patients were available for analysis. Of these, 63.4% used appointment systems to register while others chose a traditional queuing method to register. 114 telephone (30.4%) and on-site (22.9%) appointments were made, whereas other systems were less used by patients. Between the non-appointment and appointment groups there were significant differences in gender, educational degree, and residence location (P < 0.05), but no significant difference in age (P > 0.05). While the clinical appointment system had the greatest number of appointment days (25.75), the bank's self-service terminal appointment had the least number of appointment days (5.05). Leaflets sent from the hospital (50.70%) and the recommendations of friends or families (40.77%) were the two main ways of knowing about the appointment registration systems. With the exception of those who felt no need to make an appointment (30.12%), not having the capability to use the appointment systems (24.10%) and the lack of a registered health card (34.53%) were the two main reasons for not using appointment registration systems.

**Conclusions:**

Convenience was a major motivation for patients’ use of appointment registration systems. Personal knowledge and capability were the two important factors that influenced patients’ appointment system choices. Hospitals must improve the design and promotion of appointment registration systems to better facilitate their use.

## 1. Introduction

One of the greatest complaints voiced by the Chinese public has been the amount of time it takes to queue for outpatient registration in China (Y. Su, Liu, Wang, & Yi, 2006). Some patients have waited in line all night to ensure registration with a certain physician. Patients have expressed a great deal of interest in innovative appointment registration systems that could provide easier access to hospital medical services (Guo, Miao, Wei, Xing, & Zhang, 2012). Compared with the traditional queuing registration, appointment registration has many advantages. For patients, an appointment registration system offers the convenience of avoiding the long queuing time traditionally required (M. Huang, 2011). Therefore, an appointment system can significantly increase patient satisfaction with the registration process while effectively reducing the overall waiting time (Cao et al., 2011; Yin, Huang, & Huang, 2010). Such a system would also help to reduce the cross-infection that occurs during queuing registration. Hospitals can use the information that patients submit when making an appointment to follow-up after discharge (Xu & Ding, 2012; Q. X. Zhang et al., 2011). In addition, because patients must submit their personal information when making the appointment, the system would enhance the consistency and continuity of patient medical records (Liu et al., 2009). Some appointment systems could offer even greater convenience, such as the use of mobile phones to send real-time reminders or notifications of emergency consultation time changes (Chen, Tu, Xiong, & Xu, 2012; Guy et al., 2012). For hospitals, appointment systems can optimize medical procedures, realize outpatient shunts and reduce economic costs (X. M. Huang, 2011; Luo, Zeng, & Huang, 2011; J. Zhang, 2010). In the past decade, appointment registration systems have become increasingly important in hospitals that are seeking to increase efficiency and decrease operational costs.

Given their many advantages, hospitals are experimenting with novel appointment registration systems such as mobile phone, web-based, bank-hospital cooperation, clinical and others (Cheng, 2011; Ji, Lv, & Xue, 2009; Meng, Xu, Xu, Li, & Wu, 2012). Although increasingly diversified appointment registration systems are being introduced, hospitals remain essentially uninformed about patients’ opinions of the various systems. Therefore, the aim of this study was to investigate the use of diversified appointment registration systems and explore the factors influencing patients’ registration system choices.

## 2. Methods

### 2.1 Study Setting

Located in Chengdu City, Sichuan Province, the West China Hospital (founded in 1892) is one of China's leading general hospitals. In 2011, it was ranked second in the Best Service Hospital of China category and provided outpatient services to 3,554,895 patients. Facing the growing pressure of patient demand, the West China Hospital had strong motivation to improve its appointment registration systems to increase its efficiency.

Since 1996, the West China Hospital has experimented with various appointment registration systems. Now, it operates six system types.

- The on-site appointment system (OAS) requires that patients come to the hospital and queue at the appointment window to make the appointment.
- The 114 telephone appointment system (114) is based on the 114 information directory assistance platform, which is very familiar to the Chinese public as an information query platform.
- The 95533 telephone appointment system (95533) is based on China Construction Bank's customer call center.
- The clinical appointment system (CAS) allows doctors to help patients make an appointment for the next meeting immediately after their consultation.
- The bank self-service terminal appointment system (BST) is based on the self-service terminal in the bank.
- The web-based appointment system (WAS) uses the hospital's website.

Patients can use their citizen ID cards to apply for ‘Health Card’. Only patients with West China Hospital's ‘Health Card’, can use all of these systems. Without the Health Card, patients can obtain a temporary card that requires on-site appointment registration.

### 2.2 Data Collection

A questionnaire survey was used to collect the data. The survey was approved by the West China Hospital's outpatient department in advance. The study participants were outpatients who were randomly recruited from West China Hospital in February 2012. The questionnaires were distributed to outpatients waiting for consultation or those who had just finished their consultation. Informed consent was obtained from the participants before questionnaire distribution, and the completed questionnaires were collected at once. Outpatients under 18 years old completed the questionnaire with their guardians’ help. A total of 1,150 questionnaires were distributed and 1,009 valid questionnaires were collected, for a response rate of 87.74%.

The demographic information for each respondent including gender, age, educational degree and home residence, was collected. The respondents were then divided into two groups depending on whether they chose an appointment system or the traditional queuing method to register. For the appointment group, we collected information on which specific appointment system they used, the number of days in advance that they made the appointment and how they had learned about the available systems. For the queuing group, the reason for not using the appointment registration systems was collected. In addition, multiple choice questions were designed to determine how the respondents had learned about the registration systems and their reasons for not using them.

### 2.3 Statistical Analysis

We used SPSS 19.0 for Windows for all statistical analyses. Continuous variables are presented as means and standard deviations. Categorical variables are presented as frequencies and percentages. The *t*-test and analysis of variance (ANOVA) were used to analyze continuous variables, and the chi-square test was used to analyze categorical variables. A two-tailed *P*-value < 0.05 was considered statistically significant.

## 3. Results

### 3.1 Demographic Characteristics of Respondents

As Table 1 reveals, 51.1% of the respondents were female. The average age of the respondents was 39.5, and 37.4% of them had a college education or above. The majority of the participants (95.1%) were from Sichuan Province and 61.2% were from Chengdu City.

**Table 1:** Demographic information for respondents

Variable	*n* (%)
*Gender*	
Male	493(48.90)
Female	516(51.10)
*Age,* y, mean±SD	39.5±16.010
~25	235(23.20)
26~35	202(20.00)
36~45	231(22.90)
46~55	147(14.60)
56~65	125(12.40)
66~	69(6.80)
*Educational degree*	
Primary school	138(13.70)
Middle school	261(25.90)
High school	233(23.10)
College	156(15.50)
University	221(21.90)
*Residence*	
Downtown Chengdu	358(35.50)
Suburb Chengdu	259(25.70)
Other cities of Sichuan Province	343(34.00)
Other provinces	49(4.90)

### 3.2 Respondents’ Registration Choices

As Table 2 shows, 63.4% of respondents chose to use appointment registration systems. Among these, the 114 telephone system was the favored choice (30.4%). Some still chose on-site registration (22.9%), but the other systems were scarcely used by respondents. In particular, only 0.7% of the appointment registrations were completed through the website.

**Table 2:** Registration choices of respondents

Registration choice	*n* (%)
Appointment registration	
114	307(30.4)
95533	15(1.5)
On-site appointment system (OAS)	231(22.9)
Clinical appointment system (CAS)	43(4.3)
Bank self-service terminal appointment system (BST)	37(3.7)
Web-based appointment system (WAS)	7(0.7)
Non-appointment registration	369(36.6)

### 3.3 Demographic Comparison of Two Registration Groups

There was no significant age difference between the non-appointment and appointment groups (*P* >0.05). However, the two groups were significantly different in regard to gender, educational degree, and residence location. The female respondents with higher educational degrees living in Chengdu City were more likely to use appointment systems to register (Table 3).

**Table 3:** Demographic comparison of respondents choosing appointment *vs.* non-appointment

*P* value	Appointment (*n*=640)	Non-appointment (*n*=369)	Variable
*Gender,* n (%)			0.001
Male	206(55.8)	287(44.8)	-
Female	163(44.2)	353(55.2)	-
*Age*, y, mean ± SD	38.6 ± 15.306	40.02 ± 16.391	0.174
*Educational degree,* n (%)			0.003
Primary school	53(14.4)	85(13.3)	-
Middle school	118(32.0)	143(22.3)	-
High school	68(18.4)	165(25.8)	-
College	48(13.0)	108(16.9)	-
University	82(22.2)	139(21.7)	-
*Residence location,* n (%)			0.021
Downtown Chengdu	123(33.3)	235(36.7)	-
Suburb Chengdu	81(22.0)	178(27.8)	-
Other cities of Sichuan Province	147(39.8)	196(30.6)	-
Other provinces	18(4.9)	31(4.9)	-

### 3.4 Demographic Comparison between Appointment Registration Systems

As shown in Table 4, among the respondents who chose appointment registration systems, there were no significant demographic differences in gender, age, educational degree, or residence location (*P* >0.05).

**Table 4:** Demographic comparison of appointment registration systems (N=640)

Variable	114	95533	OAS	CAS	BST	WAS	*P* value
*Gender,* n (%)							0.874
Male	136(21.25)	6(0.94)	106(16.56)	18(2.81)	19(2.97)	2(0.31)	
Female	171(26.72)	9(1.41)	125(19.53)	25(3.91)	18(2.81)	5(0.78)	
*Age,* y, mean±SD	40.37±16.24	36.4±17.44	39.87±16.85	44.93±16.48	34.62±13.46	35.86±13.15	0.100
*Educational degree,* n (%)							0.189
Primary school	37(5.78)	0	36(5.63)	7(1.09)	4(0.63)	1(0.16)	
Middle school	63(9.84)	4(0.63)	57(8.91)	9(1.41)	9(1.41)	1(0.16)	
High school	83(12.97)	3(0.47)	57(8.91)	15(2.34)	6(0.94)	1(0.16)	
College	44(6.88)	4(0.63)	39(6.09)	8(1.25)	12(1.88)	1(0.16)	
University	80(12.5)	4(0.63)	42(6.56)	4(0.63)	6(0.94)	3(0.47)	
*Residence location,* n (%)							0.199
Downtown Chengdu	113(17.66)	2(0.31)	83(12.97)	19(2.97)	17(2.66)	1(0.16)	
Suburb Chengdu	96(15.00)	5(0.78)	57(8.91)	13(2.03)	5(0.78)	2(0.31)	
Other cities of Sichuan	87(13.59)	7(1.09)	75(11.72)	9(1.41)	14(2.19)	4(0.63)	
Other provinces	11(1.72)	1(0.16)	16(2.50)	2(0.31)	1(0.16)	0	

### 3.5 Number of Appointment Days

Among the 640 respondents who used the appointment systems to register, 593 (92.66%) provided the exact number of days in advance that they had made their appointments. The one-way ANOVA showed a significant difference in the number of appointment days among the systems (*P* < 0.05). As Table 5 reveals, the clinical appointment system had the highest number of appointment days (25.75) while the BST system had the lowest number of days (5.05).

**Table 5:** Appointment days among appointment registration systems (N=593)

System	n	Mean (Day)	SD (Day)
114	304	9.17	9.60
95533	14	9.36	9.48
On-site appointment system (OAS)	191	6.39	10.03
Clinical appointment system (CAS)	40	25.75	20.37
Bank self-service terminal appointment system (BST)	37	5.05	5.65
Web-based appointment system (WAS)	7	8.86	11.74

### 3.6 Knowledge of Appointment Registration Systems

Among the 640 participants who used the appointment systems to register, 574 (89.69%) of them answered the multiple choice questions about how they had learned of the appointment systems. Hospital leaflets and the recommendations of friends or families were identified as the two main ways in which patients gathered knowledge of appointment registration systems (response rates of 50.70% and 40.77%, respectively) (Table 6). Comparatively, the Internet and TV, cooperation bank leaflets, 114 telephone recommendations and newspapers and magazines were regarded as much less effective channels for learning about the available appointment registration systems.

**Table 6:** Ways of learning about appointment registration systems (N=574)

Approaches	Rate
Internet/TV	8.89%
Newspaper/Magazine	2.96%
Recommendations from friends or families	40.77%
Leaflet from hospital	50.70%
Leaflet from cooperation bank	7.84%
114 telephone recommendations	5.57%
Others	1.92%

### 3.7 Reasons for Not Using an Appointment Registration System

Among the 369 participants who did not use the appointment systems to register, 249 (67.48%) of them provided their reasons. The most common was that they only had a temporary health card (34.54%) (Table 7). Additional factors leading to the non-use of the appointment registration systems included preferring on-site appointment (30.12%) and not knowing how to make an appointment (24.10%). An average of 5.62% of respondents thought that the current appointment registration systems were inconvenient while 3.21% reported that the 114 telephone line was always busy. An average of 1.2% of the respondents reported not knowing which department to choose, whereas 9.24% gave other reasons, such as that the decision to see a doctor was sudden or unexpected.

**Table 7:** Respondents’ reasons for not using appointment registration systems (N=249)

Reasons	Rate
Inconvenience	5.62%
114 telephone line busy	3.21%
Did not know how to make an appointment	24.10%
Did not know the correct department	1.20%
Prefer on-site appointment	30.12%
Temporary health card	34.54%
Others	9.24%

## 4. Discussion

Our study reveals that patients who understood and had the capability to use the diversified appointment registration systems had strong motivation to do so. Unlike other studies, which have reported that more people choose to register via the traditional queuing method (Ren, Tao, & Zhang, 2010; S. Su & Shih, 2003), we found that more patients used the appointment registration systems, indicating that people choose appointment registration when it offers more efficient registration methods.

Among the six systems offered by West China Hospital, the 114 telephone and on-site appointment queue were the two main systems used, with the other systems exhibiting significantly less use. As the most traditional system, on-site appointments were regarded as the most assured way to make a hospital appointment. The 114 telephone line, as a public information platform, was well-known and widely used. Compared with these two systems, the others were less familiar to the patients. All of this indicates that patients’ knowledge of appointment registration systems is an important determinant of their registration system choices.

We also found that women, people with higher educational degrees, and residents of Chengdu City tended to use the appointment registration systems. There are explanations for these three factors. First, it is a popular phenomenon in China that women take more responsibility for family life. Therefore, they usually pay more attention to medical services such as hospital appointment registration and are more familiar with the systems, thus they find it easier to understand novel appointment registration systems. Second, education could increase a patient's ability to understand and master appointment registration systems. The literature suggests that education provides people with knowledge of how to optimize the use of health services (Flouri, 2006; Ross & Van Willigen, 1997), not only by teaching people how to use medical services that already exist, but also by giving people the ability to learn new technology independently. In other words, people with higher educational degrees are more capable of using an appointment system for registration. Third, residents of Chengdu City have greater accessibility to appointment systems. Compared with patients from outside of Chengdu City, residents have more information about appointment systems. Residence also guarantees easier access to appointment systems. This combination of information and accessibility advantages results in Chengdu residents using the appointment registration systems more heavily. These significant relationships suggest that personal capability does influences whether or not to use appointment systems.

Although patients’ demographic characteristics did not differ among appointment registration systems, it is still worth noting some of the interesting findings. In particular, the patients using the traditional on-site appointment system were older than those using Internet/TV and bank-hospital cooperation systems. Some of the reasons for this phenomenon might be as follows. Because the morbidity of chronic disease increases with age, older people have a higher risk of contracting chronic diseases that require frequent doctor consultation. Thus, the older patient may prefer to make an appointment right after their consultation through the clinical appointment system with the help of the doctor. In contrast, the Internet/TV- and bank-hospital cooperation-based systems are new and foreign to most older people, whereas they are more easily accepted by younger people. Likewise, we found that BST appointments had the smallest number of appointment days while the clinical appointment system had the largest number of appointment days.

Although a previous study identified the media as an important channel for promoting appointment registration systems (L. W. Huang, 2012), this study found that hospital leaflets and recommendations from friends or families were the two most important ways that patients used to gather information about such systems, whereas fewer people turned to public media such as newspapers and magazines, or dynamic media such as the Internet and TV for information. Moreover, few people understood the informa8tion on appointment registration systems from cooperation banks, implying that patients either did not have sufficient access to appointment registration system information, or that the hospital's promotion of such systems was limited.

Despite the benefits of using appointment systems, many people still registered on-site via the traditional queuing method. With the exception of patients who did not need to make an appointment in advance, the most frequent reason for not using the appointment systems was the absence of a West China Hospital Health Card. Although patients can apply for the card for free in the hospital, it does require the patients’ presence at the hospital. Consequently, lacking a Health Card constrains patients’ use of appointment registration systems, especially for patients visiting the hospital for the first time and those from outside of Chengdu City. Meanwhile, many people did not choose appointment registration because they did not know how to use the various systems to make an appointment. Clearly, the requirement of a Health Card and insufficient knowledge for easy use result in patients avoiding appointment registration systems.

Our study helps to illuminate patients’ choices when faced with diversified appointment registration systems in an advanced medical institution, such as West China Hospital. There is potential for further investigation. First, because hospitals experiment with such systems, some of China's top hospitals are developing novel appointment systems, thus additional studies covering these innovations are necessary. Second, as one of the leading hospitals in China, West China Hospital's appointment registration systems are significantly more advanced than other small- and middle-sized Chinese hospitals. Further investigations into the use of appointment registration systems in these smaller hospitals could provide more complete information about patients’ registration choices. Thirdly, as the medical resources are not equally in all places, it is reasonable that some automatic appointment systems are not fully utilized. Further study take the patients’ equality into consideration is necessary.

## 5. Conclusions

Our findings reveal a growing demand for convenient appointment registration systems. For patients facing such systems, personal knowledge and capability are important factors that influence patients’ appointment system choices. To increase the use of such systems, hospitals must strengthen the promotion of innovative appointment registration systems. Moreover, hospitals should improve the design of such systems to make them more convenient for patients to understand and use, such as using a citizen ID card rather than the hospital's own health card. More personalized design and promotion might increase the use of appointment registration systems, benefit patients, and improve hospital efficiency.

## References

[ref1] Cao W. J, Wan Y, Tu H. B, Shang F. J, Liu D. H, Tan Z. J, Sun C. H, Xu Y (2011). A web-based appointment system to reduce waiting for outpatients: a retrospective study. BMC Health Services Research.

[ref2] Chen J. H, Tu S. G, Xiong H, Xu N. Z (2012). Practice of mobile phone real-name system appointment registration. Chinese Nursing Research.

[ref3] Cheng N (2011). Design and realization of SMS application in hospital. Computer Era.

[ref4] Flouri E (2006). Parental interest in children's education, children's self-esteem and locus of control, and later educational attainment: Twenty-six year follow-up of the 1970 British Birth Cohort. British Journal of Educational Psychology.

[ref5] Guo R, Miao Z. M, Wei G, Xing S. C, Zhang Y (2012). The present situation and suggestions of the domestic appointment registration of hospital. Progress in Modern Biomedicine.

[ref6] Guy R, Hocking J, Wand H, Stott S, Ali H, Kaldor J (2012). How effective are short message service reminders at increasing clinic attendance? A meta-analysis and systematic review. Health Services Research.

[ref7] Huang L. W (2012). The practice and exploration of appointment registration service for outpatients in modern hospital. Chinese Journal Clinical Rational Drug Use.

[ref8] Huang M (2011). Exploration and practice about outpatient appointment registration service mode. Gansu Journal of Traditional Chinese Medicine.

[ref9] Huang X. M (2011). Analysis of the application of web appointment registration in hospital. Modern Hospital.

[ref10] Ji L, Lv J. W, Xue W. G (2009). Development and application of SMS platform for appointment registration. Chinese Medical Equipment Journal.

[ref11] Liu L. S, Yao Z, Ji X. M, Liu D. H, Zhang Y, Fei X. Y (2009). Status of outpatient registration and appointment service in Beijing and its process investigation. China Journal of Modern Medicine.

[ref12] Luo X. B, Zeng F, Huang H (2011). Process optimization of hospital outpatient patient flow. China Medical Education Technology.

[ref13] Meng J, Xu L, Xu J, Li X. F, Wu D (2012). The research on the process optimization of outpatient department based on one-stop service under the cooperation of bank and hospital. Chinese Hospitals.

[ref14] Ren Y. J, Tao S. L, Zhang C. Y (2010). Status analysis on service category registration appointed in public hospitals. Hospital Administration Journal of Chinese People's Liberation Army.

[ref15] Ross C. E, Van Willigen M (1997). Education and the subjective quality of life. Journal of Health and Social Behavior.

[ref16] Su S, Shih C. L (2003). Managing a mixed-registration-type appointment system in outpatient clinics. International Journal of Medical Informatics.

[ref17] Su Y, Liu J. L, Wang Y, Yi X. M (2006). The idea about the mode of a patient- centered modern clinic. Journal of Medical Postgraduates.

[ref18] Xu Y. X, Ding H. X (2012). Current situation analysis of the hospital outpatient appointment services. Clinical Medical Engineering.

[ref19] Yin X. Q, Huang Z. Z, Huang L. J (2010). Design and research on appointed outpatient registration system in modern hospital. Hospital Digitalization.

[ref20] Zhang J (2010). Online booking application of registration service processes. Computer and Digital Engineering.

[ref21] Zhang Q. X, Wang H. M, Zheng C. J, Chen N. T, Guo G. X, He S. N (2011). The practice and innovation of self-help registration and appointment registration for outpatients. Journal of Traditional Chinese Medicine Management.

